# Functional hypothalamic amenorrhea in adolescent athletes impairs bone accrual and increases fracture risk

**DOI:** 10.3389/fendo.2025.1709695

**Published:** 2025-12-09

**Authors:** Laurel Wong, Lily Leibner, Camila Vicioso, Bina Shah, Sheena C. Ranade

**Affiliations:** 1 Leni and Peter W. May Department of Orthopaedic Surgery, Icahn School of Medicine at Mount Sinai, New York, NY, United States; 2 Raquel and Jaime Gilinski Department of Obstetrics, Gynecology and Reproductive Science, Icahn School of Medicine at Mount Sinai, New York, NY, United States; 3 Jack and Lucy Clark Department of Pediatrics, Icahn School of Medicine at Mount Sinai, New York, NY, United States

**Keywords:** functional hypothalamic amenorrhea (FHA), adolescent athletes, hypoestrogenism, bone density, female athlete triad, relative energy deficiency in sport (RED-S)

## Abstract

Functional hypothalamic amenorrhea (FHA) is a reversible neuroendocrine condition prevalent among adolescent female athletes that often results from energy deficiency, reflecting an imbalance between energy intake and expenditure due to factors such as disordered eating, psychological stress, and excessive physical activity. By disrupting hypothalamic-pituitary-ovarian (HPO) axis signaling, FHA in adolescence typically leads to hypoestrogenism and subsequent impairment of bone mineral accrual during a crucial period of skeletal development. This review synthesizes current evidence on the pathophysiology of FHA in relation to bone health, emphasizing the impact of altered estrogen, IGF-1, leptin, and cortisol levels. We further summarize the main risk factors of FHA and examine their effect on reduced bone mineral density (BMD), compromised bone microarchitecture, and increased fracture risk. Studies emphasize the high risk of osteopenia, osteoporosis, and stress fractures in female athletes with FHA. Diagnosis of FHA requires exclusion of organic pathology and a multidisciplinary evaluation of orthopedic, nutritional, endocrinological, psychological, and exercise-related contributors. Evidence-based management prioritizes lifestyle modification, nutritional rehabilitation, and psychological support, with transdermal estrogen therapy as a promising treatment for refractory cases. Ongoing controversies include the limited skeletal benefits of oral contraceptives versus growing evidence for transdermal estrogen, and the paradoxical effects of exercise as both protective and harmful under energy-deficient conditions. Additionally, persistent clinical challenges are highlighted, such as underdiagnosis of menstrual dysfunction and lasting microarchitectural deficits despite weight restoration. Ultimately, early identification and intervention are essential to optimize long-term skeletal and reproductive outcomes for adolescent female athletes affected by FHA.

## Introduction

Functional Hypothalamic Amenorrhea (FHA) is the cessation of menses due to suppression or insufficiency of the hypothalamic-pituitary-ovarian (HPO) axis in the absence of any identifiable organic causes (1). FHA results from low energy availability, an imbalance between energy intake and expenditure, which may stem from factors such as anorexia nervosa (AN), excessive exercise, weight loss, or psychological stress (2). Adolescent female athletes are particularly vulnerable to developing FHA due to high physical and psychological demands as well as decreased overall energy availability. In fact, studies indicate that approximately 54% of adolescent athletes under 18 years old experience menstrual dysfunctions such as FHA as opposed to 21% in the equivalent non-athlete population (3).

Adolescence and young adulthood are critical periods for optimizing long-term bone health, underscoring the importance of proper nutrition and healthy hormonal regulation in this time period. Since estrogen plays a crucial role in maintaining bone health, hypoestrogenism in FHA due to HPO axis dysregulation can have a detrimental effect (4). Adequate nutrition is required to maintain energy balance as well as hormonal and long-term bone health in female athletes who have heightened energy demands (5). When FHA occurs during adolescence, it can interfere with peak bone mass acquisition, leading to persistent deficits in bone density which can double the risk of fractures later in life (6). This disruption of skeletal homeostasis is evident through reductions in bone mineral density (BMD). Lu et al. shows that approximately 15% of patients with FHA (average age: 24.64 ± 6.02 years) experience low bone mass, which is correlated with hypoestrogenism and weight loss (7). Defined by the American College of Sports Medicine in 1992, the “female athlete triad” describes this phenomenon, highlighting the interplay between low energy availability and bone health (8). Ultimately, this decrease in BMD increases the risk of osteopenia and osteoporosis, heightening fracture susceptibility (9). The concept of Relative Energy Deficiency in Sport (RED-S), introduced and most recently updated in the 2023 International Olympic Committee (IOC) Consensus Statement, expands upon the Female Athlete Triad by recognizing the broader multisystem consequences of low energy availability across both sexes and all levels of sport participation. RED-S emphasizes that inadequate energy intake relative to expenditure affects not only reproductive and skeletal health, but also cardiovascular, metabolic, and psychological function (10, 11). Given these risks, providers must use multidisciplinary approaches to identify bone health issues in female athletes during key growth periods.

This review aims to define the effects of functional hypothalamic amenorrhea (FHA) on female bone health, emphasizing timely recognition and referral. This review will provide evidence-based recommendations for physicians and healthcare providers to address underlying hormonal disruptions contributing to FHA. Additionally, we include a discussion of current and emerging treatment strategies, highlight gaps in the literature, and identify areas of future research for FHA management in young female athletes.

## Pathophysiology

### Overview of bone development in adolescent populations–hormonal perspectives

Bone accrual during adolescence is critical, with up to 90% of peak bone mass achieved by the end of puberty (12). Adolescence represents a time with high bone-turnover, where bone formation and resorption levels peak before decreasing to adult levels in late puberty. These metabolic processes throughout puberty are intricately regulated by the balance of gonadal hormones, growth hormones (GH), and insulin-like growth factor-1 (IGF-1) (13).

### Estrogen

Estrogens, particularly estradiol, play pivotal roles in regulating bone turnover and growth plate closure (14). Estradiol has a biphasic effect on bone growth, serving distinct functions at different stages of adolescence (15). In early adolescence, it stimulates longitudinal bone growth by upregulating osteoblast activity and promoting osteoclast apoptosis (16). As estradiol levels rise in late adolescence, its role shifts toward promoting epiphyseal plate closure. Estradiol acts directly on the growth plate chondrocytes to accelerate their maturation and increase the expression of estrogen receptor-α (ERα), causing cessation of growth plate cartilage production (17).

The HPO axis is a complex endocrine system that controls the development and regulation of the female reproductive system (18). First, in the hypothalamus, neurosecretory cells secrete gonadotropin-releasing hormone (GnRH) in a pulsatile manner (19). This stimulates gonadotropes in the anterior pituitary gland to secrete follicle-stimulating hormone (FSH) and luteinizing hormone (LH), which act on the ovaries to stimulate follicular development, ovulation, corpus luteum formation, and the production of estrogen and progesterone (20). These ovarian hormones then exert feedback control on the hypothalamus and pituitary to regulate the cycle. This process becomes fully active at the time of puberty.

In FHA, low energy availability resulting from inadequate caloric intake relative to energy expenditure, whether due to risk factors such as restrictive eating, high training load, or psychological stress, suppresses GnRH pulsatility, thereby disrupting the HPO axis (21). This disturbance leads to reduced LH and FSH secretion which results in decreased ovarian estrogen and progesterone production, anovulation, and amenorrhea. Estrogen deficiency accelerates bone resorption by impairing osteoblast function and removing estrogen’s inhibitory effect on osteoclasts, creating an imbalance in bone remodeling that favors net bone loss (22). Furthermore, Kisspeptin, an endogenous hormone that stimulates GnRH secretion, is modulated by signals of energy status such as leptin, ghrelin, and insulin (23). In a female athlete with depleted energy stores, Kisspeptin secretion may be reduced, further causing dysregulation of the HPO axis and FHA (24).

### Growth hormone

In addition to estrogen, GH also plays a direct role in growth plate maturation (25). IGF-1, primarily stimulated by GH in the liver, enhances osteoblast activity and promotes chondrocyte proliferation and differentiation at the growth plates, leading to longitudinal bone growth (26). Throughout adolescence, GH and IGF-1 work synergistically with estradiol to regulate epiphyseal plate closure (27). However, FHA leads to suppressed GH-IGF signaling, primarily due to reduced hepatic IGF-1 production despite normal or elevated GH levels, indicating GH resistance. This state of GH resistance exacerbates bone loss by impairing osteoblast function and reducing bone matrix production, ultimately compromising peak bone mass acquisition. The concurrent hypoestrogenism caused by FHA further amplifies bone resorption, reducing BMD. This disruption of the GH-IGF axis increases the risk of osteopenia, osteoporosis, and stress fractures, with lasting consequences for skeletal integrity (2).

### Leptin, cortisol, and thyroid hormones

Leptin is a hormone released by adipose tissue that regulates energy balance and appetite, and plays a key role in the hypothalamic regulation of reproductive function (28). Low leptin levels signal to the brain that energy stores are insufficient, leading to the suppression of the hypothalamic-pituitary-gonadal axis and reduced estrogen production (21). In female athletes with low body fat, leptin levels are reduced and thus contribute to FHA. Additionally, elevated cortisol and thyroid levels, both commonly seen in FHA due to chronic stress or energy deficits, contribute to bone loss by increasing osteoclastic activity and inhibiting osteoblast function (29). This hormonal excess in FHA leads to lower bone mineral density and an increased risk of fractures (30).

## Risk factors for functional hypothalamic amenorrhea

FHA in adolescent females is a multifactorial condition that arises from an intricate interplay of physiological, psychological, and behavioral stressors (**Table 1**). The primary contributors to FHA include disordered eating patterns, excessive exercise, psychological stress, and low body fat, all of which can have profound implications for bone health and overall well-being (31).

**Table 1:** Risk factors for FHA and clinical implications on bone health.

Risk factor category	Description	Mechanisms	Clinical implications on bone health	Treatment
Eating Disorders	Anorexia nervosa, restrictive or purging behaviors leading to energy deficiency	Sustained energy deficiency suppresses GnRH signaling, disrupting the HPO axis, leading to hypoestrogenism. GH resistance, hypercortisolemia, and alterations in adipokines further exacerbate bone loss	Lower BMD, impaired bone microarchitecture, osteopenia, osteoporosis, increased fracture risk, increased stress fracture risk	Nutritional rehabilitation, psychotherapy (CBT/FBT), weight and BMI monitoring, endocrine referral
Excessive Exercise	High-intensity or high-volume training leading to increased energy expenditure without adequate intake			Reduce training intensity, increase caloric intake, multidisciplinary approach (sports medicine, nutrition, endocrinology)
Psychological Factors	Anxiety, depression, body dysmorphia, psychological stress	Chronic stress increases TNF-a, increasing RANKL signaling. Concurrent activation of the HPA axis increases cortisol and metabolic adaptation, suppressing GnRH signaling		Stress management, psychological counseling, treatment of anxiety/depression, psychiatric referral if needed
Low Body Fat	Low caloric intake and increased activity leading to energy deficiency	Reduced leptin and kisspeptin suppress GnRH signaling		Weight restoration, nutritional support, monitor body composition, psychological counseling

### Eating disorders and anorexia nervosa

One of the fundamental risk factors for FHA is an imbalance between energy intake and expenditure; adolescent females who engage in restrictive eating behaviors, such as anorexia nervosa (AN), are at high risk (32). AN is defined by severe restriction of caloric and nutrient intake relative to requirements, intense fear of gaining weight, and distorted body image, leading to chronic energy deficiency and low weight (<18.5 BMI) (33). This sustained caloric and energy deficit induces a survival mechanism whereby the body aims to conserve energy during starvation by shutting down non-essential functions, including reproduction. Therefore, patients with AN are prone to having suppressed GnRH pulsatility and a disrupted HPO axis, thus leading to hypoestrogenism. Additional hormonal changes in AN, such as growth hormone resistance, hypercortisolemia, and alterations in adipokines such as low leptin levels, further exacerbate bone loss (21).

Studies support this by indicating that young women with eating disorders experience significant skeletal deficits, including reduced bone accretion and increased risk of fractures. Faje et al. reported that adolescent females ages 14–22 years old with AN had lower areal bone mineral density at the lumbar spine and hip as well as impaired cortical and trabecular microarchitecture, indicating lower bone strength (34). Moreover, a long-term population-based cohort study of 193 young women who acquired AN during puberty observed a three-fold higher risk of fractures as far as 38 years after diagnosis, and a 57% cumulative incidence risk of any fracture (35).

Furthermore, Kandemir et al. illustrate that both female athletes with AN and those with secondary amenorrhea exhibit lower BMD compared to healthy controls, with more severe bone defects observed in AN (36). The study also highlights that AN patients have greater impairments in trabecular bone microarchitecture, including lower trabecular number and thinner trabeculae. Additionally, AN patients exhibited lower bone formation markers, further indicating diminished overall bone health. Recent research underscores that female athletes with eating disorders should meet established weight and health criteria before resuming full training regimens to mitigate the risk of severe skeletal consequences (37).

### Excessive exercise

The number of adolescent girls participating in competitive sports has increased substantially over the past several decades, largely due to the implementation of Title IX in 1972 (38). While this has led to numerous physical and psychological benefits, it has also been accompanied by a rise in cases of the female athlete triad, a syndrome which encompasses one or more interrelated conditions including low energy availability, menstrual dysfunction, and low bone mineral density (BMD) (39–42). In fact, one study by Skorseth et al. showed that among 38 high school distance runners, 36% had low energy availability, 54% had menstrual abnormalities, and 16% had low bone mineral density (BMD) (43).

Low energy availability resulting from high training volumes or intensities without adequate caloric intake plays a central role in disrupting the HPO axis. Exercise alone does not cause FHA; rather, it is the imbalance between energy intake and expenditure that leads to hypothalamic suppression, estrogen deficiency, and menstrual irregularities (44–46). A retrospective study by Ackerman et al. of 175 young athletes ages 14–25 found that athletes with amenorrhea have four times the lifetime fracture risk compared to non-athletes (37). Stress fractures are particularly common in this population, occurring in approximately 32% of athletes with amenorrhea compared to only 5.9% in eumenorrheic athletes, and 0% in non-amenorrheic athletes. Furthermore, bone microarchitecture was more negatively affected in amenorrheic athletes, especially in those who sustained multiple stress fractures highlighting the detrimental combination of excess exercise with amenorrhea.

Recognizing these risks, the American College of Sports Medicine (ACSM) recommends screening for the female athlete triad and other underlying medical issues in any athlete with a history of six months or more of amenorrhea or oligomenorrhea (40). Screening includes a thorough evaluation of menstrual change, disordered eating patterns, cardiac arrhythmias such as bradycardia, weight change, depression, or stress fracture. Early intervention is crucial, as persistent energy deficiency and menstrual suppression during adolescence can have lasting consequences, increasing the risk of osteoporosis and fragility fractures later in life. Given the interplay amongst exercise, energy availability, and skeletal health, a multidisciplinary approach that includes nutrition, endocrinology, and sports medicine is essential for preventing and managing FHA in young female athletes (47).

### Psychological factors

Adolescence represents a formative stage for the development of body image and self-perception (47). During this time, individuals may be particularly vulnerable to negative body image, weight-related bullying, and excessive pressure to achieve athletic or academic excellence. These factors have been implicated in the onset and perpetuation of FHA (48).

Psychological distress is often intertwined with maladaptive behaviors such as excessive exercise and restrictive eating, which are frequently employed as coping mechanisms. However, rather than alleviating stress, these behaviors often serve as amplifiers, perpetuating a cycle of anxiety and physiological dysregulation (49). Mood disorders, such as anxiety and depression, are frequently associated with amenorrhea, and behaviors like hyper-exercise and restrictive eating may reflect underlying obsessive or anxious tendencies (50, 51). A meta-analysis conducted by Morrison et al. found that psychological factors associated with FHA include depression and preoccupied attitudes surrounding eating, specifically driven by a desire for thinness (21). Women with FHA demonstrated higher levels of anxiety, sleep disorders, dysfunctional attitudes, and alexithymia compared to women without FHA. Given this interplay, a comprehensive psychological assessment is essential, and in cases where these symptoms are identified, referral to therapeutic and psychiatric care is recommended (52).

Beyond its impact on menstrual function, psychological stress exerts direct effects on skeletal health. Chronic stress is associated with low-grade inflammation, as evidenced by increased levels of proinflammatory markers such as tumor necrosis factor-alpha (TNF-α), which upregulate receptor activator nuclear factor kappa-B ligand (RANKL) signaling and promote bone resorption (21). Additionally, stress-induced hyperactivation of the sympathetic nervous system leads to elevated noradrenaline levels, which negatively impact bone metabolism (53). Osteoclasts and osteoblasts express adrenergic receptors, and studies suggest that stress-induced bone loss can be mitigated with beta-adrenergic antagonists such as propranolol (54). These findings highlight a direct biological link between psychological stress and skeletal deterioration.

Furthermore, the hypothalamic-pituitary-adrenal (HPA) axis plays a pivotal role in the intersection of psychological stress, energy metabolism, and reproductive suppression (55). Psychological stressors, whether external (i.e., academic pressure, familial pressure, athletic demands) or internal (i.e., fear of weight gain, low self-esteem, obsessive thinking, emotional distress, perfectionism), activate the HPA axis, leading to increased cortisol secretion and metabolic adaptations that ultimately suppress GnRH pulsatility (56). Both human and animal studies suggest that energy imbalance sensitizes the HPO axis to psychological stress, heightening its vulnerability to menstrual disturbances (57, 58). Importantly, not only actual stressors but also the perception or anticipation of stress can elicit similar endocrine disruptors, reinforcing the complex bidirectional relationship between psychological and metabolic stress in FHA (59).

Addressing the psychological underpinnings of FHA is crucial not only for restoring menstrual function but also for safeguarding long-term bone health in young female athletes.

### Low body fat

Low body fat is an independent yet closely intertwined risk factor for FHA in adolescent females. Adequate fat mass is essential for normal pubertal development and menstrual regulation, as adipose tissue functions not only as a reservoir for energy but also as an active endocrine organ (21). Specifically, adipose tissue produces the hormone leptin, which serves as a gauge for energy reserves and guides the central nervous system to balance food intake and energy expenditure accordingly (60).

Evidence suggests that the minimum level of body fat percentage to initiate menarche is approximately 20%, and between 17-22% is required to maintain normal menstruation (61). Latzer et al. reported that a total body fat percentage of 21.2% had the highest discriminative ability for the resumption of menses in adolescents with FHA, with a sensitivity of 88% and specificity of 85% (62).

In the context of athletics, aesthetic sports (i.e. gymnastics, cheerleading, ballet, figure skating, rhythmic gymnastics, distance running), in which leanness may confer an advantage, have a particularly high prevalence of low body fat and associated exercise-induced amenorrhea (46). A cross-sectional study conducted by Amoruso et al. of 288 female athletes under age 25 found that 41.1% of athletes practicing in aesthetic sports reported menstrual irregularities (63). Compared to athletes of non-aesthetic sports, training for aesthetic sports also significantly increased the risk of developing menstrual irregularities (64, 65). These findings demonstrate that aside from being a byproduct of disordered eating or excessive exercise, low body fat poses as a distinct physiological risk factor that can independently disrupt reproductive function through disordered metabolic and neuroendocrine signaling. The resulting hormonal changes favor bone resorption over formation, leading to reductions in bone mineral density and compromised bone microarchitecture.

### Diagnosis

The diagnosis of FHA is one of exclusion, requiring a comprehensive evaluation to rule out anatomic or organic causes of menstrual dysfunction (**Table 2**). Clinicians should consider FHA in adolescents and women with persistent menstrual cycle intervals exceeding 45 days or those experiencing absent menses for three months or more (47). A thorough clinical assessment begins with a detailed personal history, focusing on dietary habits, disordered eating behaviors, exercise patterns, weight fluctuations, stressors, mood disturbances, sleep patterns, and personality traits such as perfectionism and a high need for social approval. Additionally, a family history of eating disorders, psychiatric diagnoses, reproductive disorders, or menstrual irregularities should be obtained to identify potential genetic or environmental predispositions (1).

**Table 2:** Diagnostic components for FHA.

Diagnostic component	Relevant tests/findings
History	Menstrual history (cycle length, age at menarche, amenorrhea duration), dietary habits, disordered eating behaviors, exercise patterns, weight fluctuations, psychological stressors, mood disturbances, sleep patterns, personality traits
Physical	BMI assessment, signs of chronic illness or malnutrition, neurological findings
Pregnancy test	Serum β-human chorionic gonadotropin
Labs	Complete blood count (CBC), metabolic panel (electrolytes, glucose, renal/liver function tests, inflammatory markers), and hormonal evaluation (LH, FSH, estradiol, testosterone, prolactin, thyroid function tests, and serum cortisol, calcium, and vitamin D
Imaging	DXA scan (patients with > 6 months of amenorrhea) MRI with pituitary cuts and contrast (patients with concern for pituitary tumor)

A physical examination is essential to assess for organic causes of amenorrhea. Pregnancy must be excluded through a β-human chorionic gonadotropin test. Laboratory tests should include a complete blood count and metabolic panel, including electrolytes, glucose, renal and liver function tests, as well as inflammatory markers (i.e., erythrocyte sedimentation rate or C-reactive protein) to rule out systemic illness (47, 66, 67). Hormonal evaluation should assess levels of LH, FSH, estradiol, testosterone, prolactin, thyroid function tests, and serum cortisol, calcium, and vitamin D to rule out other endocrine imbalances contributing to menstrual dysfunction (40, 68–70).

Given the significant impact of FHA on bone health, clinicians are advised to obtain a baseline BMD measurement via dual-energy X-ray absorptiometry (DXA) in patients with six or more months of active amenorrhea (47, 71, 72). In cases where there is a history or suspicion of severe nutritional deficiency, significant energy deficit, or skeletal fragility, DXA screening may be performed earlier. MRI with pituitary cuts and contrast is recommended in adolescents with presumed FHA and a history of persistent or severe headaches; persistent vomiting that is not self-induced; changes to vision, thirst, or urination; neurologic signs; or any other clinical indicators that suggest imbalances in pituitary hormone in order to identify pituitary or other tumors (73).

In summary, in adolescent female athletes, FHA warrants careful clinical attention, as menstrual irregularities are often overlooked or misinterpreted as benign consequences of intense training. However, persistent amenorrhea reflects underlying energy deficiency and significantly increases the risk of impaired peak bone mass, osteopenia, and stress fractures. Due to the complex interplay between physiological and psychological factors, a multidisciplinary approach involving endocrinology, gynecology, sports medicine, nutrition, and mental health professionals is recommended to create a comprehensive assessment and management plan. It is vital that physicians are prepared to diagnose and comprehensively treat FHA, especially in critical periods of skeletal development.

## Treatment

### Current standard of care

Once the diagnosis of FHA is established, treatment should focus on addressing the underlying causes, such as energy deficiency, excessive exercise, and psychological stressors, rather than simply restoring menstrual cycles with pharmacologic interventions (**Table 3**) (47).

**Table 3:** Treatment options for FHA.

Treatment category	Intervention	Rationale and goals
Lifestyle Modification	• Reduce exercise intensity/duration • Ensure adequate rest and recovery	Prevent continued energy deficit
Nutritional Rehabilitation	• Increase caloric intake to 30 kcal/kg fat-free mass per day • Increase body fat percentage to > 22% • Ensure adequate dietary calcium (700 mg to 1,300 mg/day) and vitamin D (40 to 60 ng/mL) intake	Achieve positive energy availability and weight restoration
Psychological Support	• Cognitive behavioral therapy • Family-based therapy • Address underlying psychiatric conditions	Treat psychological contributors by breaking maladaptive behaviors
Hormonal Therapy (HRT)	• Transdermal 17β-estradiol (E2) therapy with cyclic oral progestin	Use in cases refractory to lifestyle and nutritional intervention

The cornerstone of FHA management is lifestyle modification. Increasing energy availability through adequate caloric intake and reducing excessive exercise are critical first steps. Nutritional rehabilitation, ideally guided by a dietitian specializing in sports nutrition or eating disorders, is essential to correcting energy deficits. This includes increasing caloric intake to reach an energy availability threshold of 30 kcal/kg fat-free mass per day. An increase in body fat percentage above 22% may be required to restore menstrual function (74). Patients should additionally receive dietary calcium (700 mg to 1,300 mg/day) and vitamin D supplementation (40 to 60 ng/mL) (75–77). A gradual reduction in training intensity, particularly for endurance athletes or those engaging in high-impact sports, may be necessary (1). Weight-bearing exercise has been shown to have beneficial effects on bone accrual and peak bone mass (78). However, research has demonstrated a persistent lack of skeletal gains and deficits to bone microarchitecture in both female athletes with eating disorders and female athletes with normal-weight amenorrhea during adolescence, despite an increase in weight-bearing exercise. Additionally, addressing psychological stressors through psychotherapy and/or medication management is recommended, particularly for patients with high levels of anxiety, depression, or disordered eating behaviors (79). Clinicians should provide comprehensive patient education regarding the recovery process, emphasizing that menstrual irregularities are expected during treatment and do not necessarily indicate failure of intervention or impaired fertility. Patients should also be counseled that ovulation may resume before the return of regular menses, meaning conception is still possible despite irregular cycles (80).

For adolescent patients with refractory FHA, hormone replacement therapy (HRT) may be indicated to address hypoestrogenic states. The American College of Obstetricians and Gynecologists recommends HRT to benefit bone health in cases of FHA that do not resolve despite lifestyle modifications aimed at restoring energy balance (46). Historically, oral contraceptive pills have been prescribed for women and adolescents with FHA to modulate endogenous hormone levels and regulate menstrual cycles; however, most studies have demonstrated limited benefit of this intervention on restoring BMD (81, 82). Instead, the Endocrine Society Clinical Practice Guideline suggests short-term HRT in the form of Transdermal 17β-estradiol (E2) therapy with cyclic oral progestin in adolescents who continue to experience FHA that is refractory to nutritional, psychological, and/or modified exercise intervention (47). E2 therapy treats FHA by replacing the estrogen deficiency that results from suppressed HPO axis activity (83). In a retrospective cohort study of patients with hypogonadism aged 15 to 24, Dural et al. demonstrated that transdermal E2 therapy had a significant benefit on bone mass acquisition at the lumbar spine (84). Unlike oral contraceptives, E2 has been shown to significantly improve bone mineral density while preserving IGF-I secretion (85). These findings support transdermal E2 as an effective strategy to improve bone accrual in this population without suppressing IGF-I.

### Future directions

A greater emphasis on precision medicine, described by tailoring interventions based on an individual’s metabolic, genetic, and psychological profile, may enhance recovery outcomes. Digital health tools, including mobile applications and wearable devices, are also being explored to monitor energy balance and menstrual cycle patterns in real-time, offering new ways to personalize treatment and track progress (86, 87).

Emerging pharmacological therapies for FHA refractory to lifestyle modification have also been under recent investigation. For example, Metreleptin is a recombinant human leptin analog that has been shown to restore menstrual function and improve markers of bone metabolism (88). However, limited data and weight loss concerns have precluded its recommendation as a standard therapy. Additionally, Kisspeptin analogs such as MVT-602 represent a promising emerging therapy for FHA, with early-phase trials demonstrating their ability to restore GnRH pulsatility and stimulate gonadotropin secretion. Although not yet available for clinical use, kisspeptin-based treatments may offer a novel, targeted approach to reactivating the reproductive axis while minimizing systemic side effects (89). Ultimately, the future of FHA management lies in a multidisciplinary, individualized approach that prioritizes long-term reproductive, metabolic, and skeletal health.

## Discussion and conclusion

FHA in adolescent female athletes poses a major risk to their musculoskeletal health, in both the short and long-term. Recognizing the breadth of risk factors that can predispose a female athlete to FHA is crucial in early detection and prevention. These risk factors include inadequate caloric intake relative to energy expenditure and psychological stressors. Orthopedists and sports medicine physicians must be prepared to diagnose and treat FHA, as adolescent female athletes may present with fractures unknowingly due to hypoestrogenism. From a preventative standpoint, these physicians may play a crucial role in identifying and addressing risk factors for FHA before long-term bone damage ensues. Understanding the interplay of hormones in the HPO axis is key in diagnosing the underlying cause of FHA and preventing a disruption in axis function. Additionally, physicians are encouraged to normalize conversations about menstruation in clinical settings across all fields, and integrate screening tools, such as period tracking apps and sleep monitors, to help athletes surveil patterns over time. Creating a multidisciplinary, athlete-centered care model that fosters open dialogue can facilitate earlier recognition of FHA and reduce long-term skeletal consequences in female athletes (90).
